# mTOR signaling in skeletal development and disease

**DOI:** 10.1038/s41413-017-0004-5

**Published:** 2018-01-30

**Authors:** Jianquan Chen, Fanxin Long

**Affiliations:** 1 Orthopedic Institute, Soochow University, Suzhou, Jiangsu 215006 China; 2 Department of Orthopaedics, The First Affiliated Hospital, Soochow University, Suzhou, Jiangsu 215006 China; 3 Departments of Orthopaedic Surgery, Medicine and Developmental Biology, Washington University School of Medicine, St. Louis, MO 63131 USA

## Abstract

The mammalian/mechanistic target of rapamycin (mTOR) is a serine/threonine protein kinase that integrates inputs from nutrients and growth factors to control many fundamental cellular processes through two distinct protein complexes mTORC1 and mTORC2. Recent mouse genetic studies have established that mTOR pathways play important roles in regulating multiple aspects of skeletal development and homeostasis. In addition, mTORC1 has emerged as a common effector mediating the bone anabolic effect of Igf1, Wnt and Bmp. Dysregulation of mTORC1 could contribute to various skeletal diseases including osteoarthritis and osteoporosis. Here we review the current understanding of mTOR signaling in skeletal development and bone homeostasis, as well as in the maintenance of articular cartilage. We speculate that targeting mTOR signaling may be a valuable approach for treating skeletal diseases.

## Skeletal development: Regulatory pathway offers drug target for bone disease

Drugs directed at a key cellular signaling pathway could prove useful for treating skeletal diseases. Jianquan Chen from Soochow University in Suzhou, China, and Fanxin Long from Washington University School of Medicine in St. Louis, Missouri, USA, provide an overview of how proteins involved in the mechanistic target of rapamycin (mTOR) signaling pathway sense and integrate a range of environmental cues to regulate bone and cartilage development. In particular, they review the differing roles of the two distinct mTOR-containing protein complexes, mTORC1 and mTORC2. Both seem to mediate bone formation and resorption but in different ways, with implications for how best to treat osteoarthritis, osteoporosis, and other degenerative skeletal diseases. The authors suggest that more specific mTOR inhibitors with minimal side effects are needed to help stimulate bone growth in these diseases.

## Introduction

The mechanistic (formerly “mammalian”) target of rapamycin, as indicated by its name, is highly sensitive to rapamycin, a drug clinically used for antifungal, immunosuppressive, and antitumor purposes. Rapamycin was initially isolated from bacteria in soil samples of Easter Island that can inhibit yeast proliferation^1^. Mechanistically, rapamycin was shown to exert its function by forming a complex with FKBP12^2^. Subsequent studies identified the targets of FKBP12-rapamycin complex in yeasts and mammals, which were named as target of rapamycin (TOR) and mammalian target of rapamycin (mTOR), respectively^3–7^. Since its discovery, extensive research over the last twenty years has indicated that mTOR pathways play important roles in regulating development and homeostasis of mammalian tissues, and that their dysregulation is implicated in pathogenesis of many human diseases.

**Fig. 1:**
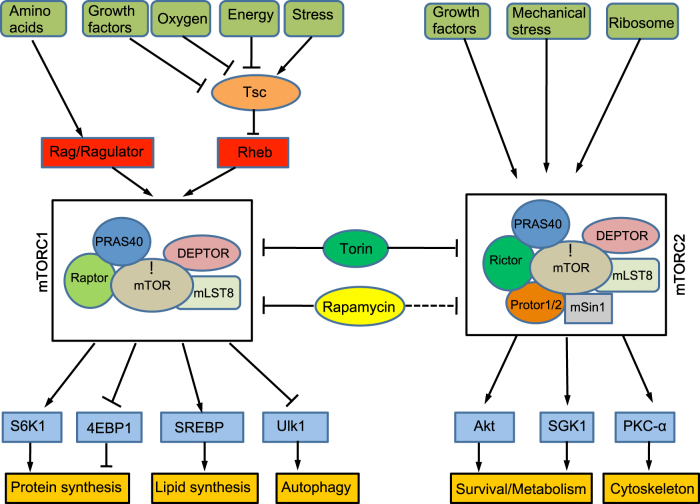
A schematic of mTORC1 and mTORC2 signaling. Dashed line denotes partial inhibition of mTORC2 by Rapamycin upon prolonged treatment

Biochemically, mTOR is an evolutionarily conserved serine/threonine protein kinase belonging to the phosphoinositide 3-kinase (PI3K)-related kinase family, and functions as a catalytic subunit in two distinct protein complexes: mTOR complex 1 (mTORC1) and complex 2 (mTORC2; Fig. 1). Initially, mTORC1 and mTORC2 were distinguished by virtue of their different sensitivities to rapamycin. Whereas mTORC1 is inhibited by acute rapamycin treatment, mTORC2 is resistant to such treatment. However, recent studies showed that prolonged rapamycin treatment also impaired mTORC2 signaling both in vitro and in vivo^8,9^. mTORC1 and mTORC2 differ in their components. While mTORC1 and mTORC2 do share two core components (mTOR, mLST8/GßL)^10,11^, they contain Raptor or Rictor as their respective unique core subunit. In addition, mTORC1 has two inhibitory subunits (PRAS40, DEPTOR)^12–15^, whereas mTORC2 contains an inhibitory subunit DEPTOR^15^ and two regulatory subunits (Protor1/2 and mSin1)^16,17^. Genetic studies revealed that ablation of mTOR blocked both mTORC1 and mTORC2 signaling whereas ablation of Raptor or Rictor only impaired mTORC1 or mTORC2 signaling, respectively^10,11^.

mTORC1 integrates a wide variety of intracellular and extracellular signals, including growth factors such as WNT and Insulin/IGF-1, the levels of oxygen, energy, stress, or amino acids, to regulate cell growth and metabolism through a number of downstream effectors^18^ (Fig. 1). One key upstream regulator of mTORC1 signaling is the Tsc1/Tsc2 complex, a GTPase-activating protein (GAP) for the small GTPase Rheb^18^. Rheb directly binds to mTORC1 and potently stimulates its activity, but Tsc1/Tsc2 negatively regulates mTORC1 by converting Rheb into its inactive GDP-bound form^19,20^. Whereas many upstream signals activate or inhibit mTORC1 activity by acting on Tsc1/Tsc2^18^, regulation of mTORC1 activity by amino acid levels is independent of TSC1/2, and instead through Rag GTPases (RagA, RagB, RagC, and RagD) and their regulators^21^. Moreover, the presence of amino acids, in particular leucine and arginine, is required for other upstream signals to activate mTORC1^18^. The lysosome has emerged as a key organelle mediating mTORC1 activation by both amino acids and growth factors. In a current model, functionally active heterodimers containing GTP-loaded RagA/B and GDP-loaded RagC/D accumulate on the cytoplasmic surface of the lysosome in response to amino acids that promote the formation of a supercomplex including the pentameric Regulator complex and the multi-subunit vacuolar ATPase complex. The active Rag heterodimer recruits mTORC1 to the lysosomal membrane where Rheb is also anchored, thus initiating mTORC1 activation. In support of the model, recent work has provided evidence that the solute carrier SLC38A9 likely functions as a sensor (“transceptor”) to arginine or glutamine concentration in the lysosome to initiate mTORC1 signaling through the Rag–Regulator complex^22,23^. Similarly, leucine stimulation of mTORC1 is dependent on the Rag GTPases but its potential transceptor in the lysosome is yet to be discovered^24^. Interestingly however, mTORC1 stimulation by glutamine appears to be independent of the Rag–Regulator complex, but requiring the small GTPase Arf1^24^. Furthermore, mTORC1 may also be activated by amino acids on the Golgi membrane where another small GTPase Rab1A recruits mTORC1 to be activated by Rheb localized in the organell^25^. Thus, the mechanisms underlying amino acid regulation of mTORC1 are undoubtedly complex and likely function in an amino acid-specific manner.

One of the major functions of mTORC1 signaling is promoting anabolic processes, including protein and lipid synthesis. The stimulation of protein synthesis is mainly through phosphorylation of p70 S6 kinase (S6K1) and eukaryotic translation initiation factor 4E-binding protein 1 (4EBP1), whereas mTORC1 activates lipid synthesis through SREBP1/2^18^. Besides its anabolic role, mTORC1 signaling inhibits catabolic processes, particularly autophagy by phosphorylating autophagy-initiating kinase Ulk1 and blocking its activation by AMPK^26^. In addition, mTORC1 has been shown to inhibit autophagy in part by inhibiting the nuclear translocation and activity of TFEB, a transcription factor important for the expression of autophagy and lysosomal genes^27^.

Similar to mTORC1 signaling, mTORC2 can be activated by various growth factors, including Wnt and Insulin/IGF1^28,29^ (Fig. 1). In addition, mTORC2 is activated by mechanical stress and ribosomes in vitro, although the molecular mechanism is still unclear^30,31^. mTORC2 controls proliferation and survival through a distinct group of downstream targets, including members of the AGC family of kinases Akt, serum and glucocorticoid-induced protein kinase 1 (SGK1), and protein kinase C-α (PKC-α)^10,11,32,33^.

## mTOR signaling in endochondral skeletal development

Mammalian bones are formed through two different mechanisms, endochondral versus intramembranous bone formation^34^. In contrast to intramembranous ossification where mesenchymal progenitors directly differentiate into osteoblasts, endochondral bone development begins with the condensation of mesenchymal progenitors due to increased cell–cell contact. Subsequently, the centrally-located cells within the mesenchymal condensations differentiate into chondrocytes, while cells at the periphery develop into the perichondrium. Following chondrogenesis, chondrocytes within the cartilage primordia initially proliferate rapidly, and then undergo a maturation process involving successive prehyertrophic, hypertrophic and terminal hypertrophic stages. Subsequently, blood vessels invade the hypertrophic cartilage and bring in progenitors for osteoclasts or osteblasts that are respectively responsible for resorbing the hypertrophic cartilage or depositing bone matrix.

Recent studies have implicated mTORC1 in regulating multiple aspects of cartilage development. Disruption of mTORC1 via deletion of Raptor in the early limb mesenchyme significantly reduced the size of limb bud cells and impaired chondrogenesis from the mesenchymal progenitors^35^. Similarly, rapamycin dramatically suppressed the formation of cartilage nodules from limb bud cells without affecting precartilaginous mesenchymal condensation^35–37^. In addition, rapamycin markedly reduced proteoglycan accumulation and the expression of chondrocyte markers in the chondrogenic ATDC5 cell line, perhaps through suppression of Sox9 expression^35,36^.

Studies of the growth plate cartilage in vivo have also revealed important roles for mTORC1 in chondrocytes. Immunofluorescence staining for phospho-S6, a common readout for mTORC1 signaling, revealed intense and nearly homogenous activity in prehypertrophic and early hypertrophic chondrocytes, but only sporadic signals in round chondrocytes^36,38^. In addition, mTORC1 was largely absent in much of the hypertrophic region except for the terminal hypertrophic chondrocytes^36,38^. Functionally, deletion of Raptor severely impaired skeletal growth through the reduction of chondrocyte size and matrix production, as well as the delay in chondrocyte hypertrophy and the eventual removal of the hypertrophic cartilage^38^. The decrease in chondrocyte size and matrix production is likely associated with the compromised protein synthesis rate in the Raptor-deficient chondrocytes. Surprisingly, ablation of Raptor did not have a major effect on chondrocyte proliferation or survival^38^. On the other hand, hyperactivation of mTORC1 signaling via Tsc1 deletion increased chondrocyte proliferation while impeding chondrocyte maturation^39^. This study further suggested that mTORC1 coordinated chondrocyte growth, proliferation, and differentiation through its downstream effector S6K1, which acts on Gli2 to stimulate transcription of parathyroid hormone-related peptide (PTHrP)^39^. However, the model is difficult to reconcile with the observation that mTORC1 activity is highest in the prehypertrophic and early hypertrophic chondrocytes but PTHrP is mainly expressed by the peri-articular chondrocytes. Moreover, global deletion of S6K1 in mice caused a milder skeletal phenotype than Raptor deletion did^40,41^. Thus, the mechanism underlying the importance of mTORC1 signaling in cartilage development remains to be fully elucidated.

In contrast to mTORC1 signaling, mTORC2 appears to play a minor role in endochondral skeletal development. Inactivation of mTORC2 signaling via ablation of Rictor only mildly affected limb growth^42^. This study further showed that deletion of Rictor did not affect chondrocyte proliferation, apoptosis, cell size, or matrix production, but instead caused a mild delay in chondrocyte hypertrophy in both embryos and postnatal mice^42^.

## mTORC1 signaling in bone formation and resorption

**Fig. 2:**
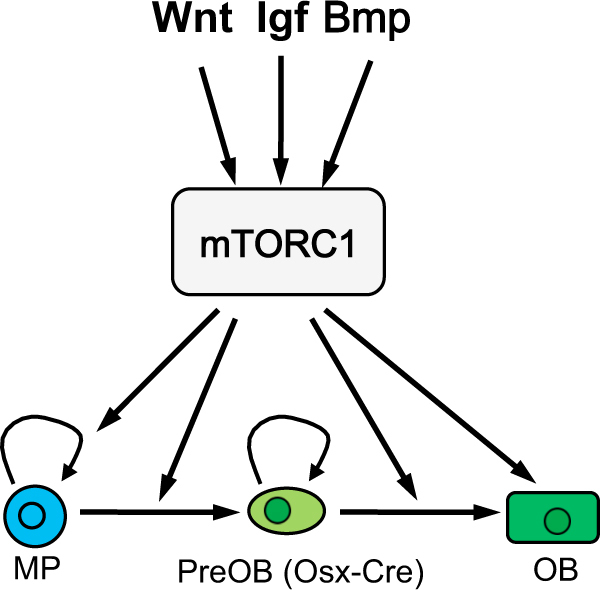
Multiple growth factors activate mTORC1 to stimulate osteoblast differentiation and activity. Curved arrows denote proliferation. MP mesenchymal progenitor, PreOB preosteoblast targeted by Osx-Cre, OB osteoblast

Bone homeostasis is maintained through the balance of bone formation and bone resorption. Osteoblasts differentiated from mesenchymal stem/progenitor cells are the chief bone-forming cells, while HSC-derived osteoclasts are responsible for bone resorption. Inhibition of mTORC1 signaling by rapamycin was shown to impair both proliferation and osteogenic differentiation of mouse bone marrow stromal cells (BMSC) in vitro, and to cause trabecular bone loss in vivo^43,44^. Conversely, activation of mTORC1 by IGF-1, an abundant growth factor present in the bone matrix and released during bone resorption, activated mTORC1 signaling to stimulate osteoblast differentiation of BMSC^44^ (Fig. 2). Similarly, bone anabolic Wnt ligands such as Wnt3a and Wnt7b have been shown to activate mTORC1 through PI3K-AKT signaling^45^. Pharmacological inhibition of mTORC1 signaling prevented Wnt7b-induced osteoblast differentiation in ST2 cells^45^. More importantly, genetic deletion of Raptor in the osteoblastic lineage cells alleviated the Wnt7b-induced high bone mass phenotype in mice^45^, indicating that Wnt7b promotes bone formation in part through mTORC1 activation. Mechanistically, mTORC1 mediates the osteogenic effect of Wnt partly by promoting glutamine catabolism and integrated stress response (ISR), which in turn induces the expression of protein anabolism genes essential for osteoblast differentiation^46,47^. In addition, Bmp2 was recently reported to induce the osteogenic program partly through a mTORC1-dependent mechanism^47^. Furthermore, Bmp signaling through Bmpr1a stimulated osteoblast activity through mTORC1 signaling in mice^48^. Thus, mTORC1 appears to be a common effector downstream of multiple bone anabolic signals.

Recent studies have further demonstrated that mTORC1 is required for the transition of preosteoblasts to mature osteoblasts^49,50^ (Fig. 2). Genetic inactivation of mTORC1 in preosteoblasts by specifically deleting Raptor in preosteoblasts with Osx-Cre caused osteopenia in mice, mainly due to a defect in bone formation. Further analyses indicated that the raptor-deficient preosteoblasts were deficient in matrix synthesis and mineralization, exhibiting a transcriptional profile of immature osteoblasts, indicative of a failure to progress beyond the early stages of osteogenesis. Interestingly, these studies showed that deletion of Raptor impaired protein synthesis without overtly affecting autophagy. Together, these findings support that mTORC1 promotes the transition from preosteoblasts to mature osteoblasts through enhancing mRNA translation. However, others reported that inhibition of mTORC1 signaling with a low dose of rapamycin enhanced preosteoblast differentiation, but prevented their proliferation in cell cultures and in mice^51^. The conflicting results from these studies could be due to the different experimental approaches. Whereas genetic ablation of Raptor with Osx-Cre inactivates mTORC1 signaling mainly in the osteoblast lineage from the preosteoblast stage onward, systemic administration of rapamycin exerts broad inhibition both within the osteoblast lineage and beyond. In addition, preosteoblasts may respond differently to the different extent of mTORC1 inhibition caused by raptor deletion versus low-dose rapamycin.

The importance of proper mTORC1 signaling in normal bone formation is further supported by the studies of the Tuberous Sclerosis (TSC) syndrome. TSC is an autosomal dominant disease with an estimated incidence of 1 in 5800 at birth and is caused by loss-of-function mutations of the *TSC1* or *TSC2* gene^52–54^. As heterodimeric TSC1 and TSC2 complex normally inhibits mTORC1 signaling by converting the active GTP-bound Rheb (a positive regulator of mTORC1) into the inactive GDP-bound form, the inactivating mutations of TSC1 or TSC2 cause hyperactive mTORC1 signaling in the TSC patients^20^. Although the main characteristics of TSC are benign tumors in skin, brain, kidney, and heart, 40–60% of the patients develop sclerotic bone lesions^55,56^. Recently, mice with TSC1 specifically deleted in neural crest cells were shown to exhibit sclerotic craniofacial bone lesions similar to those in TSC patients^56^. The study further revealed that deletion of TSC1 caused an expansion of osteoprogenitor cells at an early postnatal stage, leading to an increase in osteoblast number and consequently excessive bone formation. Remarkably, the sclerotic bone phenotype was completely reversed when rapamycin, a chemical inhibitor of mTORC1, was administered at an early postnatal stage, demonstrating that hyperactive mTORC1 signaling underlies the bone overgrowth caused by TSC1 deletion. In other studies, deletion of Tsc2 in mature osteoblasts or deletion of Tsc1 in preosteoblasts accelerated proliferation, but impaired osteoblast differentiation, probably through activating the STAT3/p63/Jagged/Notch pathway and suppressing Runx2^51,57^. Thus, Tsc1/Tsc2 appears to function as an important modulator for proper mTORC1 signaling to ensure a balance of osteoblast proliferation and differentiation necessary for optimal bone formation.

The exact role of mTORC1 in regulating the osteoclast lineage is controversial at present. In one study, inactivation of mTORC1 by deletion of Raptor, or hyperactivation of mTORC1 by deleting Tsc1 in osteoclast precursors with LyzM-Cre either enhanced or impaired osteoclastogenesis, respectively^58^. The study further suggested that mTORC1 inhibits osteoclast differentiation through suppression of NF-kappaB and NFATc1, both critical transcription factors of osteoclastogenesis^58^. However, a recent study showed that inhibition of mTORC1 in bone marrow macrophages by either genetic deletion or rapamycin treatment suppressed osteoclast differentiation in vitro, which was rescued by over-expression of constitutively active S6K1^59^. Moreover, mice with ablation of raptor in osteoclasts with Ctsk-Cre exhibited high bone mass phenotype due to decreased bone resorption^59^. Besides direct regulation, indirect inhibition of osteoclastogenesis and bone resorption has been reported for mTORC1 signaling in mesenchymal progenitors though not osteoblasts^60^. Therefore, mTORC1 signaling may exert stage-specific effects on the osteoclast lineage through both direct and indirect actions but a clear understanding about the roles and mechanisms warrants further investigation.

## mTORC2 signaling in bone homeostasis and osteoporosis

Like mTORC1, mTORC2 is also implicated in regulating osteoblast differentiation and function. Bone marrow stromal cells (BMSC) lacking Rictor gene exhibited reduced osteogenic potential, but an increased capacity to undergo adipogenic differentiation in vitro^30,42,61^. Similarly, knockdown of rictor in primary cultures of preosteoblasts impaired their osteogenic differentiation^62^. Interestingly, expression of Rankl, but neither Opg nor M-CSF, was significantly downregulated in Rictor-deficient BMSC, which exhibited a diminished capability to supporting osteoclastogenesis in vitro^42,63^. Thus, besides a cell-autonomous role in stimulating osteoblast differentiation, mTORC2 signaling in the osteoblast precursors also indirectly promotes osteoclastogenesis by modulating the expression of Rankl. The stimulatory effect of mTORC2 on both osteoblasts and osteoclasts helps to explain the relatively normal trabecular bone mass when Rictor was deleted in the limb mesenchymal progenitors in the mouse even though the cortical bone mass was reduced^42,63^. The differential net effect on trabecular versus cortical bone mass in those mice may be due to the more active bone resorption normally occurring in the trabecular bone. Similarly, deletion of rictor in mature osteoblasts simultaneously decreased osteoblast activity and bone resorption in the mouse, leading to notably impaired cortical bone, along with some subtle changes in the trabecular bone mass^62^.

**Fig. 3:**
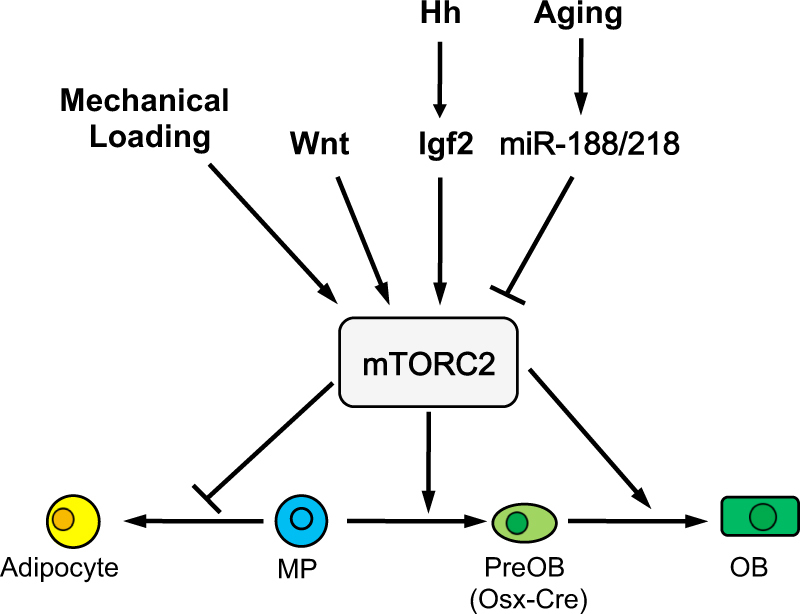
mTORC2 is a common mediator for mechanical and biochemical signals to stimulate osteoblast differentiation. mTORC2 signaling also inhibits (denoted by blocked arrow) adipocyte differentiation from bipotential mesenchyaml progenitors (MP)

mTORC2 appears to be a common mediator for both mechanical and biochemical signals to stimulate osteoblast differentiation and bone formation (Fig. 3). Rictor-deficient bones exhibited a lesser anabolic response not only to mechanical loading, but also to the anti-sclerostin antibody therapy that enhances Wnt signaling in bone^42,63^. The later finding is consistent with biochemical studies demonstrating that bone anabolic Wnt ligands such as Wnt3a, Wnt7b or Wnt10b signal through Lrp5 to activate mTORC2 and to reprogram glucose metabolism^28^. In addition, mTORC2 was shown to participate in Hedgehog (Hh)-induced osteoblast differentiation, as Hh-Gli2 signaling induced Igf2 expression that activated the mTORC2-Akt-Gli2 cascade further stimulating Hh signaling and osteogenesis^29^.

Multiple lines of evidence have implicated mTORC2 in age-related osteoporosis. Studies have shown a decrease in the expression of rictor in osteoblastic lineage cells during aging and that decreased rictor expression could contribute to the age-related switch from osteoblast to adipocyte differentiation^64,65^. Deletion of rictor in osteoblasts accelerated age-related bone loss in the mouse^64^. Interestingly, several miRNA including miR-188 and miR-218 increase with aging in either BMSC or osteoblasts, and may be responsible for the age-dependent decrease in rictor expression^64,65^. Thus, mTORC2 may serve as a potential therapeutic target for treating age-related bone loss.

## mTOR signaling and osteoarthritis

Osteoarthritis (OA) is a chronic degenerative joint disease characterized by gradual loss of articular cartilage, synovial inflammation, and subchondral bone remodeling. Recent studies have shown that mTOR was up-regulated in human OA cartilage and the articular cartilage of dogs and mice with injury-induced OA^66,67^. Moreover, activation of mTORC1 signaling via conditional ablation of Tsc1 in osteochondral progenitors with Col2a1-Cre caused spontaneous OA in mice, whereas inducible deletion of Tsc1 in chondrocytes in two-month-old mice promoted progression of aged-related and surgery-induced OA^67^. Mechanistically, activation of mTORC1 reduced expression of FGFR3 and PTH/PTHrP receptor in chondrocytes, probably through p73 and ERK1/2^67^. Conversely, inhibition of mTORC1 signaling either pharmacologically or genetically attenuated OA pathology in animal models^68–71^. In particular, systemic administration of rapamycin significantly reduced cartilage degeneration and synovial inflammation in a murine model of OA. Similarly, local administration of rapamycin through intra-articular injection inhibited chondrocyte hypertrophy and the expression of angiogenic factor VEGF by the articular cartilage in a murine injury model, therefore attenuating OA progression. Likewise, intra-articular injection of Torin 1, a potent inhibitor of both mTORC1 and mTORC2, significantly alleviated articular cartilage degeneration in a rabbit model of collagenase-induced OA partly through suppression of MMP13 and VEGF^71^. Moreover, genetic ablation of mTOR in chondrocytes reduced chondrocyte apoptosis and the expression of MMP13 in a surgery-induced OA model, thus alleviating cartilage degradation^66^. The ablation of mTOR in chondrocytes also suppressed TGF-β/Smad3 signaling in synovial tissues, thus decreasing synovial fibrosis^66^. Thus, multiple lines of evidence support the notion that hyperactive mTOR signaling contributes to OA pathogenesis.

The mechanism underlying the contribution of aberrant mTORC1 activation to OA is not completely understood. Recent studies have implicated autophagy as an important downstream mediator of mTORC1 signaling in OA pathogenesis. Autophagy is an intracellular homeostatic mechanism responsible for degrading and recycling defective macromolecules and cytoplasmic organelles, and is critical for cell survival. A number of studies showed that the expression of major autophagy markers were suppressed in human OA cartilage as well as in animal models of OA^66,72^. Moreover, inhibition of autophagy caused chondrocyte apoptosis and OA-like pathogenesis in vitro and in vivo^73,74^. Consistent with the role of mTORC1 as a major suppressor of autophagy, chondrocyte-specific activation of mTORC1 reduced the expression of key autophagy genes in the articular cartilage and caused an OA phenotype in mice^75^. Conversely, inhibition of mTORC1 signaling by either Rapamycin or Torin or by genetic deletion of mTOR in chondrocytes increased autophagy and attenuated OA progression^66,71^. Strikingly, inhibition of autophagy negated the protective effects of rapamycin on OA phenotypes^66^. Thus, suppression of autophagy in response to hyperactive mTORC1 signaling appears to be an important contributor to OA progression.

## Future directions

Despite the rapid progress in understanding the role of mTOR singaling in the skeleton, many challenges remain. For instance, the signal inputs to mTOR pathways and the corresponding mechanisms for activating mTORC1 versus mTORC2 are not fully understood^76^. Although multiple growth factors including Wnt, Igf, and Bmp can stimulate mTOR signaling in the skeleton, their relative contribution, likely dependent on the cellular context and the niche environment, is yet to be explored. Moreover, it is not clear how mTOR signaling is regulated by the nutrient status in chondrocytes, osteoblasts or osteoclasts. Acquiring such knowledge would require comprehensive biochemical studies in vitro, as well as skeleton-specific genetic studies in vivo.

It is important to identify specific downstream effector(s) mediating physiological or pathological functions of mTOR complexes in the skeleton. A recent report revealed that S6K1 only partially mediated the osteogenic effect of Wnt-mTORC1 signaling^77^. As previous work has implicated S6K1 in mediating mTORC1 signaling in aging, it would be of interest to determine whether S6K1 mediates the role of mTORC1 in the pathogenesis of OA, an age-related degenerative disease^78^. Such information could be of clinical value as specific S6K1 inhibitors have been developed and may be tested for therapeutic potentials in OA^79^.

A major challenge for targeting mTOR for therapeutic use lies with the very fact that mTOR signaling plays critical roles in many tissues and physiological processes. Although pharmacological inhibitors of mTORC1, such as rapamycin, may be adjusted to achieve partial inhibition of mTORC1 signaling, the long-term effect of mTORC1 inhibition is still uncertain. Moreover, truly specific inhibitors for mTORC1 versus mTORC2 are still lacking. Even though rapamycin is commonly considered as an mTORC1-specific inhibitor, prolonged rapamycin treatments also compromise mTORC2 signaling. Instead of directly suppressing mTOR, the future of drug development in this area may depend on tissue-specific mTOR modulators and/or process-specific downstream effectors. Identification of such modulators or effectors will also allow for development of agonists of the mTOR-dependent pathways that may be useful for stimulating bone growth in the case of osteoporosis and bone fractures.
